# Development of a Female-Targeted Lure for the Box Tree Moth *Cydalima perspectalis* (Lepidoptera: Crambidae): a Preliminary Report

**DOI:** 10.1007/s10886-019-01094-0

**Published:** 2019-08-13

**Authors:** Béla Péter Molnár, Zsolt Kárpáti, Antal Nagy, István Szarukán, Judit Csabai, Sándor Koczor, Miklós Tóth

**Affiliations:** 1 Department of Zoology, Plant Protection Institute, Centre for Agricultural Research, Hungarian Academy of Sciences, Budapest, Hungary; 2 Institute of Plant Protection, University of Debrecen, P. O. Box 400, Debrecen, H-4002 Hungary; 3 Department of Applied Chemical Ecology, Plant Protection Institute, Centre for Agricultural Research, Hungarian Academy of Sciences, Budapest, Hungary

**Keywords:** Bisexual lure, Pheromone, Flower volatiles, Trapping, Invasive pest

## Abstract

The box tree moth, *Cydalima perspectalis,* is an invasive pest in Europe causing damage on *Buxus* species. In this study, we aimed to develop a “bisexual” lure to attract both female and male moths. Based on a previous screening bioassay we tested methyl salicylate, phenylacetaldehyde and eugenol as potential attractants in different combinations. The trapping results showed that both binary and ternary blends attracted male and female moths. Catches with these blends were comparable to catches with the synthetic pheromone. Subsequently we carried out single sensillum recordings, which proved the peripheral detection of the above-mentioned compounds on male and female antennae. To identify synergistic flower volatiles, which can be also attractive and can increase the trap capture, we collected flower headspace volatiles from 12 different flowering plant species. Several components of the floral scents evoked good responses from antennae of both females and males in gas chromatography-electroantennographic detection. The most active components were tentatively identified by gas chromatography coupled mass spectrometry as benzaldehyde, *cis*-ß-ocimene, (±)-linalool and phenethyl alcohol. These selected compounds in combination did not increase significantly the trap capture compared to the methyl salicylate- phenyacetaldehyde blend. Based on these results we discovered the first attractive blend, which was able to attract both adult male and female *C. perspectalis* in field conditions. These results will yield a good basis for the optimization and development of a practically usable bisexual lure against this invasive pest.

## Introduction

The box tree moth, *Cydalima perspectalis* Walker, Lepidoptera: Crambidae (BTM) is a devastating invasive pest of the *Buxus* genus, originating in East Asia (Inoue 1982). Beside cultural, social and economic impact, the most serious threat from BTM is likely to be on the natural *Buxus* populations (Kenis et al. 2013; Mitchell et al. 2018). Boxwood is a small tree or shrub, typically growing in the understory of deciduous and evergreen broadleaved forests in south-western Europe, the Russian Caucasus and other regions (di Domenico et al. 2012; Mitchell et al. 2018). In Europe, the moth was first detected in Germany and the Netherlands (Billen 2007; Kenis et al. 2013; Krüger 2008; Straten and Muus 2010) where it was most likely introduced by plant importations. Since then it has invaded most European countries including England, France, Switzerland, Belgium, Austria, Hungary, Italy, Turkey (CABI 2017) spreading in all directions and even reaching the Ukraine and the coastal areas of the Black Sea (Nagy et al. 2017; Nesterenkova 2015).

The female-produced sex pheromone of BTM was shown to contain (*Z*)-11-hexadecenal, (*E*)-11-hexadecenal and (*Z*)-11-hexadecenol (Kawazu et al. 2007) and traps baited with a 4:1 mixture of synthetic (*Z*)- and (*E*)-11-hexadecenal proved to be useful in detecting the occurrence of the pest in Asia and in Europe (Kim and Park 2013; Nagy et al. 2017; Santi et al. 2015). The objective of the present research was to develop lures capable of attracting both males and females, rather than just males which are attracted by the sex pheromone. Tracking the seasonal activity of females could give important information about the female flight behavior and would make possible the study of physiological aspects like mating status, etc. These data would undoubtedly help to optimize management programs (Knight and Light 2005).

The first line of the study was based on observations of BTM adults readily feeding on blossoms of many herbaceous plants such as *Tagetes spp.*, *Trifolium pratense*, *Bellis perennis, Solidago x canadensis, Fallopia japonica* as well as on arboreal plants like *Buddleja davidii*, *Hedera helix*, *Rosa spp.*, *Syringa vulgaris* etc. (Molnár and Kárpáti, unpublished). The volatiles from several blossoms were collected and analyzed in an attempt to obtain floral compounds which can increase the attractiveness of BTM in the field.

The second line of this study was prompted by chance findings. In an experiment conducted on green lacewings (Chrysopidae), we were surprised to observe catches of BTM specimens at field sites far from any *Buxus* plants in traps baited with a blend of phenylacetaldehyde, methyl salicylate and acetic acid (Koczor et al., unpublished). This combination is a ternary floral attractant lure for *Chrysoperla* lacewings (Tóth et al. 2009).

Furthermore, in another field test aimed at trapping noctuids with floral attractants, a ternary blend of phenylacetaldehyde, eugenol and benzyl acetate caught significant numbers of BTM (Tóth et al., unpublished). Based on these preliminary observations, we aimed to study which of these synthetic compounds could be responsible for attraction of BTM in field conditions. Additionally, we carried out single sensillum recordings to study the ability of peripheral receptor neurons to detect these compounds.

## Methods and Materials

### Collection of Flower Volatiles

Volatile collections were conducted from freshly collected flowers of eleven arboreal plants, *Robinia pseudoacacia, Pyracantha vulgaris, Rosa spp., Philadelphus coronarius, Cornus sanguinea, Sambucus nigra, Syringa vulgaris, Ligustrum vulgare, Tilia cordata, Buddleja davidii, Hedera helix* and one perennial herb, *Asclepias syriaca*. Freshly picked flowers were placed into a glass cylinder with quick-fit connection on both ends. Charcoal filtered air was pumped at 1 l min^−1^ through the system using a vacuum pump (Thomas G 2/02 EB, Garder Denver Thomas GmbH, Fürstenfeldbruck, Germany) connected with PTFE tubes (I.D. 5 mm) to the glass cylinder. Headspace volatiles were collected on a filter containing 1.5 mg activated charcoal (Brechbühler AG, Schlieren, Switzerland) for 4 h. Before each collection the adsorbent filters were cleaned as described by Molnár et al. (2015), and after collection adsorbed compounds were immediately extracted with 40 μl of *n*-hexane into a 1.5 ml vial and kept at −40 °C. Subsequently extracts were used for electrophysiological recordings (GC-EAD) and chemical identification (GC-MS).

### Insects

For electrophysiology, box tree moths were collected at an early larval stage from public gardens in different parts of Budapest, Hungary, and kept in a climate-controlled chamber (25 ± 1 °C, 65 ± 5% RH, 16:8 h L:D photoperiod). Larvae were kept in cylindrical glass jars (internal diameter 20 cm, height 25 cm) and fed on shoots of boxwood placed in a small water container. Pupae were then collected from the shoots and placed in mesh cages until moths emerged.

### Coupled Gas Chromatography – Electroantennographic Detection (GC-EAD)

Analyses using GC-EAD were performed on an Agilent 6890 N gas chromatograph (Agilent Technologies Inc., Santa Clara, CA, USA) equipped with two HP-5 capillary columns (30 m × 0.32 mm × 0.25 μm, J&W Scientific, Folsom, CA, USA). Aliquots of floral collections (2 μl) were injected in splitless mode (230 °C, split opened after 30 s), and the oven temperature was held at 35 °C for 1 min, and then increased at a rate of 10 °C min^−1^ up to 230 °C. Carrier gas was helium at a constant flow rate of 45 cm sec^−1^. The GC effluent from both columns was split equally in a low dead volume glass four-way splitter. Two pieces of deactivated fused silica capillary columns (100 cm × 0.32 mm) were connected to the four-way splitter. One lead to the FID (280 °C) and the other lead to the heated (230 °C) EAD transfer line (Syntech, Kirchzarten, Germany) and into a glass tube (ID 10 mm) through which charcoal-filtered and humidified air (1 l/min) was passed over the antennal preparation.

The antenna of a 1–2 d old female BTM was excised and inserted into glass capillary filled with Ringer solution and attached to the reference silver/silver chloride electrode. The tip of the antenna was cut and inserted into the recording glass electrode filled with Ringer solution. The antennal signal was amplified 10 times, converted to a digital signal (IDAC-2, Syntech), and recorded simultaneously with the FID signal using GC-EAD software (GC-EAD 2014, vers. 1.2.5, Syntech).

### Coupled Gas Chromatography - Mass Spectrometry (GC-MS)

Flower volatile samples were analyzed by GC-MS using a HP Agilent 5890 GC and 5975 MS (Agilent Technologies) equipped with a HP-5 UI capillary column (30 m × 0.25 mm × 0.25 um, J&W). Injection was in splitless mode (250 °C, split opened after 30 s) and the oven temperature was maintained at 35 °C for the first 3 min, then increased at 10 °C min^−1^ to 240 °C and held for 10 min. Carrier gas was helium at constant flow rate of 36 cm sec^−1^, the ionization voltage was 70 eV, and scanning was *m/z* 29–300 with 2 scan/s. Compounds were tentatively identified by matching their mass spectra with those in the MS Libraries (NIST 11 and Wiley) using the Software ChemStation (D.01.02.16), and identifications were verified by injection of synthetic reference compounds. Retention indices were calculated using C8-C40 alkane calibration standard and compared to those in the libraries.

### Single Sensillum Recordings (SSR)

Single sensillum recordings were performed according to the procedure described by Kárpáti et al. (2013). For the recordings, a 1–4 d old unmated female or male was inserted into a plastic pipette tip to immobilize the body. The head protruded from the tip and the antennae were placed on a glass slide covered with inert glue (Tanglefoot, Planet Natural Ltd., Bozeman, MT, USA). A sharpened tungsten wire reference electrode was inserted into the abdomen. The sensilla on the immobilized antenna were localized under a light microscope (Olympus BX51WI) at 750x magnification. The electrolytically-sharpened tungsten recording electrode was inserted into the base of the sensillum using a micromanipulator (DC-3 K, Märzhäuser-Wetzlar GmbH & Co Kg, Wetzlar, Germany). The extracellular analog signal was 10x amplified using a pre-amplification probe (Universal Single Ended AC/DC Probe PRS-1, Syntech). The amplified signal was filtered with 50–60 Hz suppression and sampled with the rate of 96,000 sample/s using integrated digital-analog converter (IDAC-4, Syntech) connected to a computer. The antenna was maintained under a charcoal filtered and humidified air stream (1 l min^−1^).

The synthetic odors were diluted in *n*-hexane and 10 μl of the corresponding dilutions (10, 100, 1000 and 10,000 ng/μl) of the compounds were deposited on a filter paper disk (12.7 mm Ø; Schleicher & Schnell GmbH, Dassel, Germany), which was then placed into a Pasteur-pipette and used as a stimulus cartridge. As a blank stimulus 10 μl of *n*-hexane was used. The 0.5 s long stimuli (0.5 l min^−1^) were delivered into the continuous air stream (1 l min^−1^) using a stimulus controller (CS-55, Syntech). The action potentials were recorded for 10 s, starting 2 s before the stimulus onset. The spikes were counted manually 1 s before and 1 s after the stimulus onset. The spike number of pre-stimulus represents the spontaneous activity of the neuron. The spike frequency was calculated as the number of spikes during the stimulus time (1 s) minus the number of spikes before the stimulus onset (1 s) and expressed as the number of spikes per seconds.

### Field Trapping Tests

Field trapping tests were conducted at several sites in Hungary, using standard methods (Roelofs and Cardé 1977). Traps were arranged in blocks so that each block contained one trap of each treatment. Traps within blocks were separated by 8–10 m, and blocks were at least 30 m apart. Traps were inspected at intervals, typically twice weekly, when captured insects were recorded and removed.

Traps used were funnel traps (CSALOMON® VARL+; Plant Protection Institute (PPI), CAR HAS, Budapest, Hungary; www.csalomontraps.com) as used previously for trapping several large moth species (Subchev et al. 2004; Tóth et al. 2000, 2010). Vaportape® strips (Hercon Environmental Inc., Emigsville, PA, USA) were placed in each trap bucket to kill captured moths.

Candidate floral lures were formulated in polyethylene bag dispensers (PE bag) consisting of the undiluted compound (100 μl) loaded onto a piece of dental roll (1 cm; Celluron, Paul Hartmann Ag., Heidenheim, Germany) in a polyethylene bag (1.0 × 1.5 cm × 0.02 mm thick) made by heat sealing polyethylene lay-flat tubing. The dispensers were attached to plastic strips (8 × 1 cm) for ease of handling and wrapped singly in aluminum foil and stored at −18 °C until used. In the field, lures were changed at four-week intervals, as previous experience with similar baits showed that they may start to lose activity after this period (Tóth et al. 2012, 2017). Chemicals were obtained from Sigma-Aldrich Kft, Budapest, Hungary, and were > 95% pure as stated by the supplier.

Pheromone lures were prepared by loading 100 μg of a 4:1 blend of (*Z*)- and (*E*)-11-hexadecenal (purity >98% by GC) onto red rubber 11 mm sleeve stopper dispensers (Wheaton Co., Millville, NJ, USA). Pheromone lures were replaced at 4 week intervals. BTM caught were identified and separated by gender based on morphological features described by Mally and Nuss (2010).

Statistical analyses of catches were conducted with the software packages StatView® v4.01 and Super ANOVA® v1.11 (Abacus Concepts Inc., Berkeley, CA). As even transformed data did not meet the assumptions of a parametric test, the non-parametric Kruskal-Wallis test was used. When the Kruskal-Wallis test indicated significant differences, pairwise comparisons by Mann-Whitney U test were conducted.

#### Field Experiment 1

The aim of this field test was to confirm previous chance captures of BTM described in the Introduction, and to test the relative importance of the addition of eugenol and methyl salicylate to phenylacetaldehyde. The test was run in Northeastern Hungary (near Nyíregyháza, Szabolcs-Szatmár-Bereg county) during 23 May – 18 October 2016 with five replicate blocks. Treatments were: phenylacetaldehyde + eugenol; phenylacetaldehyde; phenylacetaldehyde + eugenol + methyl salicylate; synthetic BTM pheromone; unbaited.

#### Field Experiment 2

This test aimed to determine the attractiveness of a blend of floral volatiles shown to have electrophysiological activity, alone and in combination with a blend of methyl salicylate and phenylacetaldehyde found to be attractive to BTM in previous, non-targeted trapping bioassays. The test was run in Nagykovácsi, (Pest Ccounty, Hungary) from 4 to 28 September 2015 with five replicates blocks. Treatments were: benzaldehyde + cis-ß-ocimene + (±)-linalool + phenylethylalcohol (FLO); phenylacetaldehyde + methyl salicylate (PAA + SAL); phenylacetaldehyde + methyl salicylate + benzaldehyde + cis-ß-ocimene + (±)-linalool + phenethyl alcohol (PAA + SAL+ FLO); synthetic BTM pheromone (Pher); unbaited.

## Results

### Identification of Electrophysiologically-Active Compounds Emitted by Flowers

**Fig. 1:**
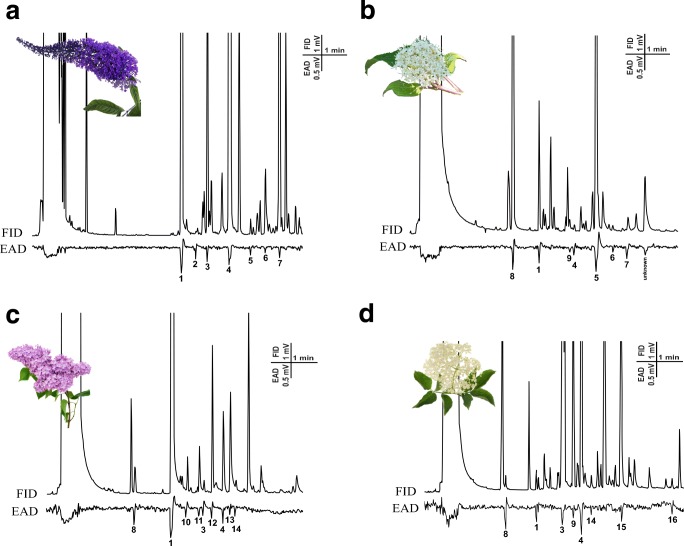
Representative GC-EAD traces of female box tree moths, *Cydalima perspectalis,* to flower volatile collections, top, GC trace (FID); bottom, antennal signal (EAD). **a***Buddleja davidii* (**b**) *Cornus sanguinea* (**c**) *Syringa vulgaris* (**d**) *Sambucus nigra.* Electrophysiologically-active compounds are numbered: 1) benzaldehyde, 2) ß-myrcene, 3) *cis*-ß-ocimene 4) linalool 5) methyl salicylate 6) eugenol 7) geranyl acetone 8) 2-heptanol 9) linalool oxide 10) D-limonene 11) *trans*-ß-ocimene 12) acetophenone 13) 4-oxoisophorone 14) *cis*-citral 15) ß-citronellol 16) ß-caryophyllene

**Fig. 2:**
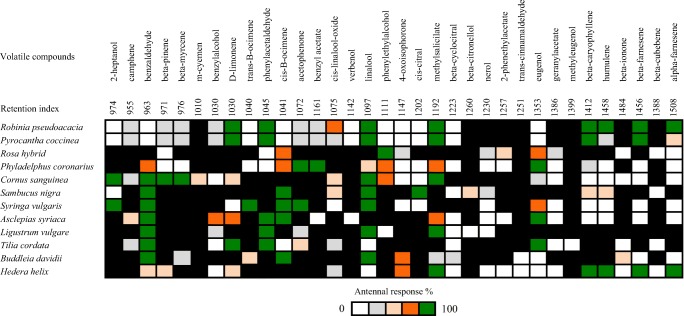
Heatmap of GC-EAD recordings from female *Cydalima perspectalis* antennae to flower volatile compounds of volatile collections from 12 different plant species. Colours indicate increasing response percentage from low (white) medium (orange) to high (green), black indicates the lack of certain compounds from the flower scent. Retention Indices were recorded on a HP-5 GC column

Based on our previous observation of BTM adults feeding on different flowers, we collected headspace volatiles from these flowers and analyzed them by GC-EAD and GC-MS to detect and identify antennally-active compounds. Headspace volatiles from fresh flowers of 12 plant species were collected, and representative GC-EAD traces are shown in Fig. 1. In total, 34 compounds elicited EAD responses and were identified as candidate attractants (Fig. 2). Compounds which elicited the highest responses and were present in volatiles from at least five of the tested flower species were selected for field testing. These were benzaldehyde, *cis*-ß-ocimene, linalool and phenylethylalcohol.

### Peripheral Detection of Compounds by SSR

**Fig. 3:**
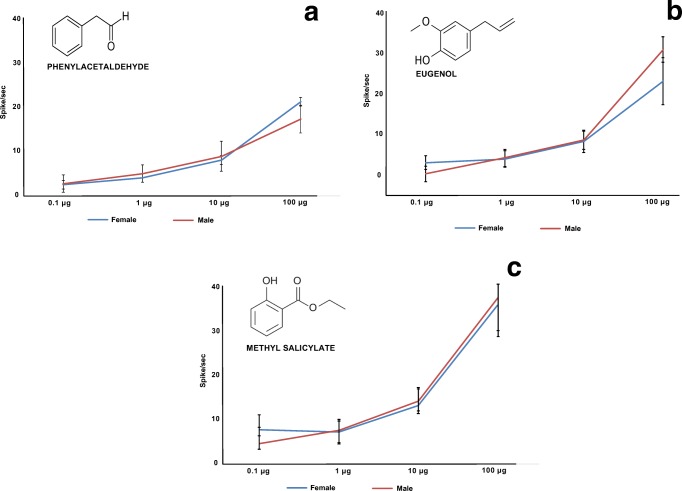
Dose–response curves of single sensillum recordings on *sensilla basiconica* (*N* = 17) of male and female *Cydalima perspectalis* antennae to phenylacetaldehyde, eugenol and methyl salicylate (average number of spikes of the olfactory sensory neuron response)

Single sensillum recordings were obtained to characterize the response of the olfactory sensory neurons (OSNs) tuned to eugenol, methyl salicylate and phenylacetaldehyde. *Sensilla basiconica* and *sensilla trichodea*, located on the 4th–10th segments of the antennae, were involved in the recordings, and only *s. basiconica* responded to the odors tested. In all, 88 contacts were established on 5 male and 5 female antennae. Out of the 88 recordings, in 17 on males and 20 on females, *s. basiconica* gave positive responses to eugenol, phenylacetaldehyde and methyl salicylate. We did not find any significant differences between the responses of males and females to the odors tested (Fig. 3). In both sexes, methyl salicylate gave the strongest response at the two high doses (10 and 100 μg). The spontaneous activity of the tested neurons varied between 15 to 18 Hz. Reduced spike frequencies compared to the spontaneous activity, which indicates inhibitory responses, were not found either during or after the stimulation onset.

### Field Trapping Experiments

In Experiment 1, phenylacetaldehyde alone caught some moths, but the catch was not significantly different from that in unbaited traps. Traps baited with the binary combination of phenylacetaldehyde + eugenol or the ternary combination of phenylacetaldehyde + eugenol + methyl salicylate caught more than unbaited traps and the catches were not significantly different between each other, although the latter treatment caught numerically most moths.

**Fig. 4:**
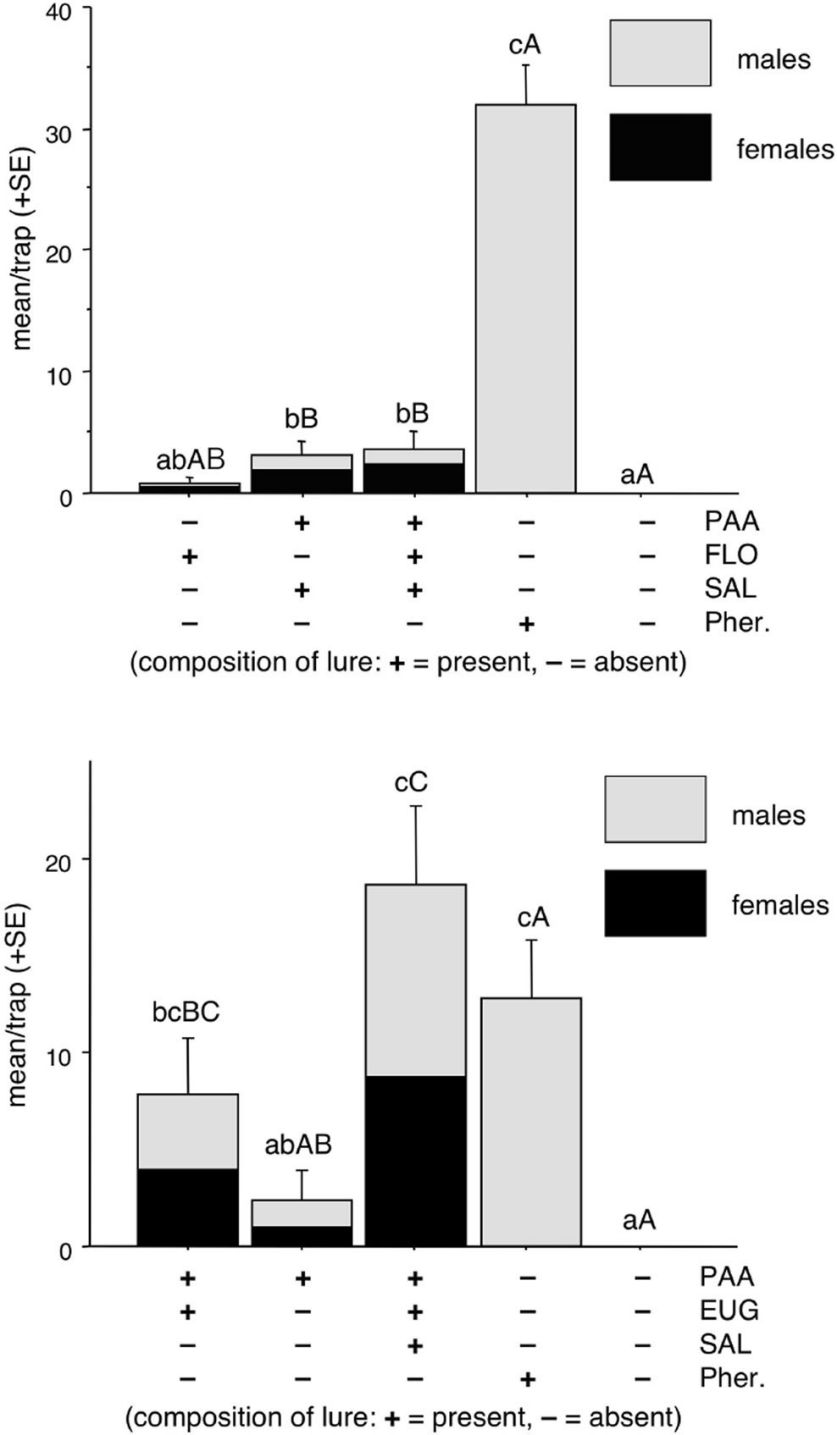
Mean catches of *Cydalima perspectalis* in traps baited with phenylacetaldehyde (PAA), binary mixture with eugenol (EUG), its ternary mixture with eugenol plus methyl salicylate (SAL) or synthetic pheromone (Pher.) in field experiment 1. Columns with same letter (upper case: female catches, lower case: both sexes together) are not significantly different at *P* = 0.05 by Kruskal-Wallis followed by pairwise comparisons with Mann-Whitney test

Traps baited with synthetic pheromone or unbaited traps caught no female BTM, and the catch of females in the traps baited with phenylacetaldehyde (PAA) alone did not differ significantly from the unbaited treatment (Fig. 4). Both treatments containing phenylacetaldehyde together with eugenol caught significantly more females than unbaited or pheromone alone. Catches of both sexes showed a similar trend, except that the pheromone attracted only males and more than any of the other treatments (Fig. 4).

**Fig. 5:**
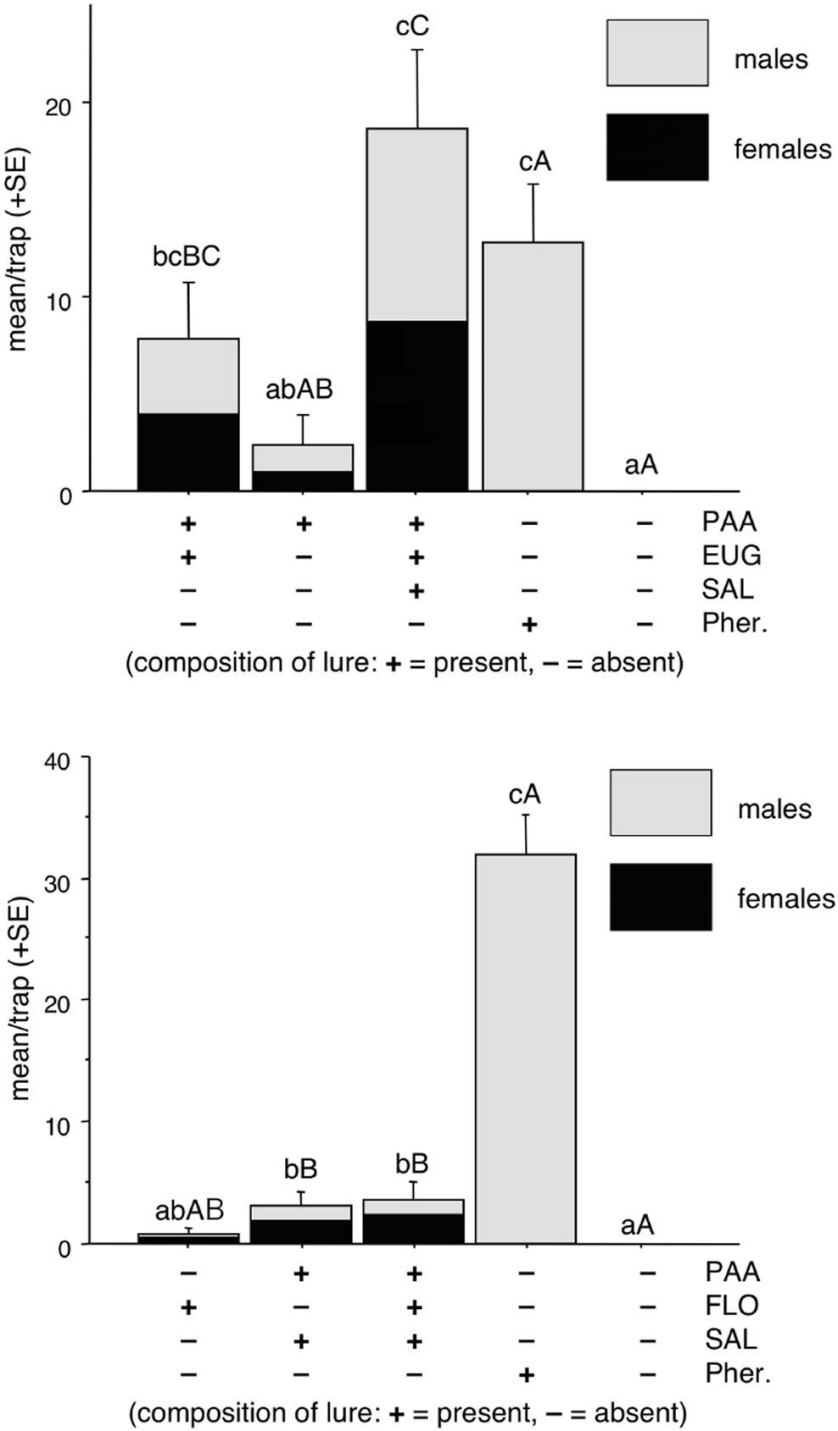
Mean catches of *Cydalima perspectalis* in traps baited with phenylacetaldehyde (PAA), its binary mixture with methyl salicylate (SAL), its multicomponent mixture with methyl salicylate plus synthetic floral mixture (FLO; benzaldehyde, *cis*-β-ocimene, (±)-linalool, phenethyl alcohol), the floral mixture (FLO) alone or synthetic pheromone (Pher.) in field experiment 2. Columns with same letter (upper case: female catches, lower case: both sexes together) are not significantly different at *P* = 0.05 by Kruskal-Wallis followed by pairwise comparisons with Mann-Whitney test

In Experiment 2, all traps baited with floral compounds (FLO) captured both males and females (Fig. 5). Traps baited with the binary mixture of phenylacetaldehyde + methyl salicylate or the binary mixture plus floral odors caught more than unbaited traps. Catches in these two treatments were similar, and also did not differ from catch in traps baited with the floral odors only. The latter did not catch more than unbaited traps. Highest catches were recorded in traps baited with the pheromone, although these were all males.

Significant numbers of non-target moth species were caught during the trapping bioassays and these are shown in Table 1.

**Table 1:** Total numbers of non-target moth species caught with different lures during field experiments 1 and 2

Compound	Lure composition						
BTM pheromone	**+**						**–**
Phenylacetaldehyde		**+**	**+**	**+**		**+**	**–**
Eugenol			**+**	**+**			**–**
Methyl salicylate				**+**		**+**	**–**
Floral odor					**+**	**+**	**–**
Family/Species	Number of moths captured						
Noctuidae							
*Abrostola triplasia* L.	0	13	41	41	0	0	0
*Autographa gamma* L.	0	12	43	72	0	3	0
*Diachrysia chrysitis* Aukt.	0	2	9	11	0	0	0
*Helicoverpa armigera* Hbn.	1	19	22	28	2	0	0
*MacDunnoughia confusa* Steph.	0	26	58	47	0	0	0
Pyralidae							
*Haritala ruralis* Scop	8	42	77	76	4	18	0
*Palpita vitrealis* Rossi	0	2	1	6	0	0	0

## Discussion

In the present study, we demonstrated attraction of male and female BTM moths to a ternary blend of phenylacetaldehyde, eugenol and methyl salicylate. Phenylacetaldehyde is recognized as broadly attractive to Lepidoptera (Cantelo and Jacobson 1979; Creighton et al. 1973; Meagher 2001), although in the case of BTM this compound alone was only weakly attractive. Future fine-tuning will be necessary to determine the relative importance of eugenol and methyl salicylate in the ternary blend for optimizing BTM catches.

We performed SSR experiments to understand the selectivity and sensitivity of the OSNs tuned to eugenol, methyl salicylate and phenylacetaldehyde. In both sexes only *sensilla basiconica* located on the antennae responded to all three compounds indicating that BTM is able to detect these odors at the sensory level. Also, in other moth species ORNs located in *sensilla basiconica* detect plant volatiles (Kafka 1987; Pophof et al. 2005). We found only broadly tuned OSNs responding to the tested odors even in low concentrations (100 ng, 1 μg). This result is consistent with earlier studies where generalist OSNs were found responding to plant volatiles (Andersson et al. 2009; de Bruyne and Baker 2008; Ignell and Hansson 2004). Female chemosensilla detecting frass volatiles of conspecific larvae of BTM have also been described (Molnár et al. 2017). OSNs tuned to these compounds have been verified in other insect species (Bengtsson et al. 2009; Binyameen et al. 2014; Hallem and Carlson 2006; Münch and Galizia 2016).

Based on the previous observations of BTM males and females feeding on flowering plants we hypothesized that the volatile components of flowers may be more attractive than the previously used ternary blend. Therefore, we collected volatiles from these flowers and identified electrophysiologically active volatile compounds which potentially could increase the attractiveness. Out of 34 compounds we selected the compounds which elicited the highest responses and were present in volatiles from at least five of the tested flower species. We also found methyl-salicylate, eugenol and phenylacetaldehyde in the headspace volatiles of some tested flowers, although they did not elicit strong electrophysiological responses except for phenylacetaldehyde. However, the four components (benzaldehyde, cis-ß-ocimene, linalool, phenethylalcohol) which were used in the second field test are attractive floral compounds for other Lepidopteran species. For instance, the ternary mixture of linalool, benzaldehyde and benzylalcohol attracts the sphinx moth (*Manduca sexta*) (Riffell et al. 2009). Also, linalool in combination with other floral components attracts many moth species (Meagher and Landolt 2008). *cis*-ß-Ocimene, in combination with other floral odors can attract cotton bollworm (*Helicoverpa armigera*) females in the wind tunnel (Bruce and Cork 2001). The alfalfa looper moth (*Autographa californica*) can be attracted in the field by phenethylalcohol also in a mixture with other components (Landolt et al. 2001). Although these floral components can attract other moth species they did not increase the trap catches of BTM alone or in combination with phenylacetaldehyde and methyl salicylate in our second trapping bioassay. This could be due to either the incorrect ratio of the components in the blend or some missing key components which did not elicit strong electrophysiological response but are essential for attraction. Further experiments were carried out, but numbers of BTM caught were low and erratic because of declining populations. In Central Europe, the boxwood is not a native species and is only present as a popular ornamental shrub. Since introduction of BTM their larvae have destroyed *Buxus* stands in private and public gardens, cemeteries etc. in the region and BTM populations have decreased dramatically in the absence of host plants.

Non-target noctuids caught during the trapping bioassays all belong to the Plusiinae and Melicleptriinae subfamilies, which are known to respond to phenylacetaldehyde (Pawar et al. 1983; Plepys et al. 2002; Tóth et al. 2010). Catches of *Autographa gamma* and *MacDunnoughia confusa* can be increased by the addition of eugenol and benzyl acetate (Tóth et al. 2019). Tendencies in field experiment 2 may confirm this suggesting that the addition of eugenol may be beneficial. Among the Pyralids *Haritala ruralis* is known to respond to floral blends containing phenylacetaldehyde (Tóth et al. 2018). The few specimens of *Palpita vitrealis* caught may also be due to the presence of phenylacetaldehyde.

It is of great advantage that the present bisexual BTM lure is catching also females. Protandry is a well-known phenomenon in case of insects (see e.g. Muralimohan and Srinivasa 2008), and it could be observed well with traps baited with a lure capable to attract both male and female specimens. The difference of the flight of females vs. males may have great significance in pest management. Timing of the insecticide sprays to the flight of females could be more precise as it probably correlates better to egg laying than catch patterns of males recorded in pheromone traps (Knight and Light 2004, 2005).

Also, the trapping or baiting of females versus males may have a more direct negative impact on pest populations. Preliminary demonstrations have been made of lure-and-kill approaches using lures containing phenylacetaldehyde plus other floral compounds against pest noctuids (Camelo et al. 2007; Landolt et al. 1991). The development of lure-and-kill technology against BTM using the bisexual lure developed on the basis of results in this study could provide an alternative to insecticide cover sprays on box trees that are damaged, reducing both pesticide amounts used and pesticide contact with the ornamentals.

It is not known whether the sex ratio of BTM captured in traps baited with floral compounds mirror the natural sex ratio of the local population. The sex ratios of catches with the three different floral lures were similar, but future studies on whether the female percentages in the catch represent the actual sex ratio or are biased for some reason should be a priority for better management of BTM.

Finally, we anticipate that the ternary bisexual attractant described in this study will serve as a good basis for further lure optimization which would result in a practically usable lure for both sexes of BTM.
